# Emergency planning and management in health care: priority research topics

**DOI:** 10.1057/hs.2013.15

**Published:** 2013-11-22

**Authors:** Alan Boyd, Naomi Chambers, Simon French, Duncan Shaw, Russell King, Alison Whitehead

**Affiliations:** 1 Manchester Business School, The University of Manchester, Manchester, U.K.; 2 University of Warwick, Coventry, U.K.; 3 Royal Free London NHS Foundation Trust, London, U.K.; 4 Wrightington, Wigan and Leigh NHS Foundation Trust, Wigan, U.K.

**Keywords:** health care, emergency planning and management, business continuity, research priorities, conceptual model

## Abstract

Many major incidents have significant impacts on people's health, placing additional demands on health-care organisations. The main aim of this paper is to suggest a prioritised agenda for organisational and management research on emergency planning and management relevant to U.K. health care, based on a scoping study. A secondary aim is to enhance knowledge and understanding of health-care emergency planning among the wider research community, by highlighting key issues and perspectives on the subject and presenting a conceptual model. The study findings have much in common with those of previous U.S.-focused scoping reviews, and with a recent U.K.-based review, confirming the relative paucity of U.K.-based research. No individual research topic scored highly on all of the key measures identified, with communities and organisations appearing to differ about which topics are the most important. Four broad research priorities are suggested: the affected public; inter- and intra-organisational collaboration; preparing responders and their organisations; and prioritisation and decision making.

## Introduction

Most disasters and many major incidents have significant short- and long-term impacts on people's health, placing additional demands on health-care organisations (Pan American Health Organization, 2000). Emergency planning and management to address these demands are complex. The many determinants of mental and physical health mean that a wide variety of non-health-care organisations may have roles in prevention and recovery efforts,^1^ spanning the public, private and voluntary sectors. During the response phase, temporary organisational networks need to be deployed rapidly, but with large-scale emergencies not respecting administrative boundaries, even the health-care organisations involved in the care of casualties requiring urgent treatment may not be used to working together with such tight coordination or short timescales. Depending on the nature and severity of the hazard, specialist equipment and resources that are in short supply or geographically distant may also need to be mobilised, posing significant logistical issues. In some disasters, entire health-care facilities may be put out of action or overwhelmed, necessitating fundamental changes to care processes and standards for a period.

In addition to planning for emergency incidents, emergency planners in health-care organisations are also likely to be involved in business continuity planning and management. This is an important role, as the health-care sector is a major component of the economies of developed nations (OECD, 2011). Good access to efficient, high-quality health care is a high priority for societies and their political representatives, so incidents that adversely affect everyday services can have serious reputational and financial consequences for organisations, in both the public and private sectors, through mechanisms such as penalty clauses in contracts, adverse media coverage and litigation. People receiving health-care services, particularly acute hospital care, are already in ill health, making them particularly vulnerable if the service they rely on is affected by an incident. Thus, for example, fires in hospital buildings, where patients are typically elderly, lack mobility and may require a sterile environment or electrical equipment for their treatment, pose difficult challenges for safe evacuation (Wapling *et al*, 2009).

The environment for health-care emergency and business continuity planning is also continually changing, with factors as varied as climate change, technological advances in medical care, an ageing population, economic cycles and the reorganisation of services potentially having an impact. Indeed, during 2010–2011, impending changes to health-care emergency planning arrangements in England, and uncertainty about their exact nature and timing and about the level of commitment to emergency planning, were of great concern to staff interviewed as part of the study. Given such complexity and change, there is an ongoing need for high quality organisational and management research that does not presume a well-ordered, rational world, where the development of utility-maximising tools to be applied by planners and managers is sufficient, but engages with the messiness and politics of organisational life in order to provide a strong foundation for policy and practice (Sementelli, 2007). Such research may, however, be difficult to conduct because of planners concerns about security, or the rapid onset of many emergency incidents, which does not sit easily with slow moving research ethical approval processes. Careful planning of research is therefore also important, so that it properly addresses knowledge gaps of practical significance and has the necessary support in place to facilitate access to the field.

The main aim of this paper is to suggest a prioritised agenda for organisational and management research on emergency planning and management relevant to U.K. health care based on a scoping study commissioned by the National Institute for Health Research. A secondary aim is to enhance knowledge and understanding of health-care emergency planning among the wider research community by highlighting key issues and perspectives on the subject and presenting a conceptual model. Stuart-Black *et al* (2008) provides an overview of health-care emergency planning in the United Kingdom. While the primary focus was on the United Kingdom, comparisons were also made with the United States, because much of the research on emergency planning and management has been conducted there, and because planned changes to the NHS in England being proposed by the government at the time of the study appeared to be moving towards a more market-based health-care system closer to that in the United States. In the first section below, a conceptual model of health-care emergency planning is presented in order to further elaborate and communicate the topic. The following section describes the methods that were used in order to gather information about potential research topics and to prioritise them. The findings of the study are then presented in the form of clusters of topics and a discussion about their importance.

## A health-care perspective on emergency planning and management

Drawing on a U.S. definition of public health preparedness planning (Nelson *et al*, 2007), and the U.K. NHS definition of a major incident (Department of Health, 2005), health emergency planning can be defined as:

*A coordinated, cyclical process of planning, implementation, evaluation and learning which aims to increase the capability of society to prevent, protect against, respond to, and recover from any occurrence which presents a serious threat to the health of the community, or disrupts the health care system, or causes (or is likely to cause) such numbers or types of casualties as to require special arrangements to be implemented by one or more health care organisations.*

**Figure 1:**
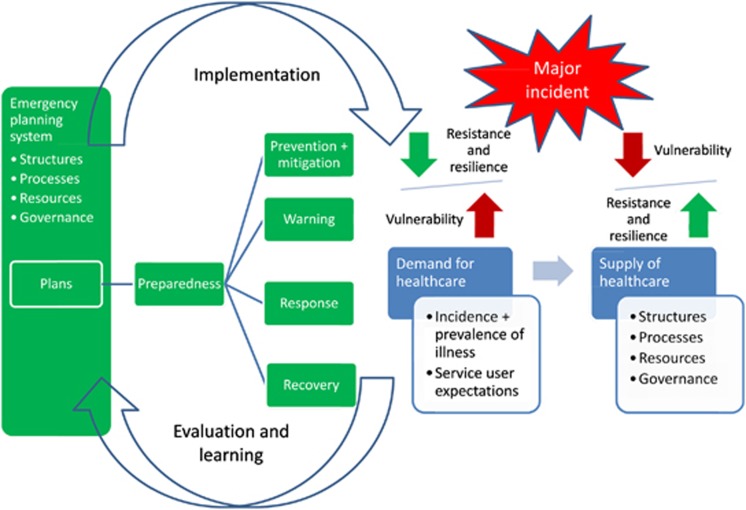
Conceptual model of health emergency planning.

At the start of the study, a conceptual model of health emergency planning was developed based on this definition and on a preliminary scan of the literature, which highlighted the importance of mismatched resources and demands (Dombrowsky, 1998), the nature of the hazard and the capacities of organisations and communities (Wisner *et al*, 2004), and various activities that need to be planned for and managed (Hodgkinson & Stewart, 1991; Pearson & Mitroff, 1993). The model shows from an organisational perspective the key processes that are involved and the connections between them, and is represented schematically in Figure 1. An incident may increase demand for health care, or reduce its supply, or both. The increase in demand may have two aspects – the simple volume of patients, and also the nature of the health problems they present. Similarly, the supply of health care, which relies on a range of structures, processes and resources including human resources, facilities, organisation, equipment and supplies, has both quantitative and qualitative aspects. During a radiological incident, for example, the available staff may be unfamiliar with the symptoms of radiation poisoning and what the appropriate treatment procedures are. It is these quantitative and qualitative mismatches between demand and supply, which vary according to the nature of the incident and the vulnerability to it of the demand and supply systems, that can compromise the quality or efficiency of care.

A variety of potential hazards need to be planned for. Health-care organisations have major roles to play in preventing, mitigating and responding to pandemic human disease, such as influenza. This is both the highest impact risk on the U.K. National Risk Register matrix (Cabinet Office, 2010) and also one of the most likely risks to occur. While demand increases, supply of health-care staff can also be reduced, most obviously because they are infected, but also for a variety of other reasons, including caring for children if schools are closed, caring for ill relatives and the impacts of the pandemic on public transport systems.

It is characteristic of disasters that they reduce the supply of health care through their adverse effects on general infrastructure, so it is important that that business continuity plans are integrated into wider emergency plans. Similarly, loss of staff and supply chain planning are key components of pandemic flu planning. Nevertheless, business continuity issues usually occur in relation to relatively small-scale incidents. NHS emergency planners spend most of their time on such issues, yet the concept of business continuity planning is fairly new to the NHS, with the first mention being made in 2005 (Department of Health, 2005) and further interim advice provided in 2008 (Department of Health, 2008). Intelligence gathered from study interviewees, workshop participants and the networks of research team members suggests that formal, in-depth business impact analysis is not generally conducted by operational units within NHS organisations due to competing pressures on managers' time and the lack of specialist support – in many organisations one person takes responsibility for both emergency planning and business continuity.

Severe weather such as snow, extreme cold, heat waves and storms resulting in flooding can produce significant short- and long-term demand for health care, with, for example, flooding causing psychosocial health problems (Du *et al*, 2010), and heat waves leading to cases of sunburn, heat exhaustion, respiratory problems and other illnesses associated with the hot weather, such as food poisoning (Department of Health, 2010). But severe weather may as much affect the supply of health care through its effects on general infrastructure, again bringing it into the domain of business continuity planning. Further internally focused planning may also be required because existing NHS patients in both community and institutional settings are among groups vulnerable to adverse health impacts from severe weather. For example, large buildings such as hospitals may struggle to keep room temperatures down during heat waves and to prevent patients from becoming dehydrated. Such internally focused planning may be regarded as part of business continuity planning, but business continuity planning also needs to be outward looking, engaging external suppliers, for example.

Terrorist attacks, while thankfully rare in the United Kingdom, can place massive short-term demands on ambulances and hospitals, and then a large task to provide follow-up psychological care. For example, the 7/7 terrorist attacks on London in 2005 resulted in 56 deaths, including the 4 suicide bombers, and over 700 injured within a single day, with blast injuries not commonly encountered by health-care staff. Major health-care incidents involving chemical, biological, radiological or nuclear (CBRN) materials, whether from industrial accidents or acts of terrorism, are also relatively rare, but are likely to be difficult to manage, partly on account of that rarity, despite significant investment by the NHS in preparing for CBRN incidents. Although ambulance services and acute hospitals have protective and decontamination equipment, staff may lack the training to use them properly, for example. Finally, major transport accidents seldom require more than a local response, other than access to existing specialist regional trauma and burns injury centres, but significant additional demands can be placed on local ambulance services and hospitals, with follow-up psychosocial care being commonly required.

In general, emergency planning aims to increase the resistance and resilience of health-care supply and demand systems by implementing measures to prevent incidents, and preparing systems to respond to and recover from the incidents that do occur. To achieve this, an emergency planning system needs to have structures, processes, resources and governance that enable it to develop suitable plans, and to implement those plans effectively. It also needs to be able to continuously improve plans through conducting regular exercises and drills and learning from them (Nelson *et al*, 2007). As disasters may occur when events contradict accepted assumptions (Turner & Pidgeon, 1997), a ‘double loop learning' system (Argyris & Schön, 1996; Herzog, 2007) is needed.

This conceptual model provides an overview of health emergency planning and helps to clarify the relationship between health-care emergency planning and business continuity planning. The understanding generated by developing the model informed subsequent data collection and analysis undertaken in the remainder of the study. For example, although reports of major incidents may give some insights into business continuity plans that may have been invoked as part of the response, unpublished internal reports also need to be accessed in order to provide a full picture. The concept of supply and demand *systems* highlights the need not just to focus on NHS organisations and their internal workings, but also to bring in external partner organisations and suppliers, and to go beyond organisations by taking a public health perspective that includes communities and their roles. Governance arrangements are also relevant, not only for emergency planning, but also for the health care and other systems that are involved, because they influence how organisations act. Consideration of learning processes is important, and not just for the purposes of identifying policy and practice issues and research gaps. It also provides a means of understanding how research can have an impact on the practice of emergency planning, which is crucial. There is little point in applied research that is subsequently ignored by practitioners!

## Methodology, data collection and analysis

The study aimed to identify future research priorities across a wide, complex area of policy and practice, spanning different hazards, organisations and sectors. In view of this, and the limited resources available for the study, it was therefore appropriate to conduct it as a scoping study, which describes the breadth and key characteristics of research on a topic rather than performing in-depth analyses of individual pieces of research. Scoping study methodology is underdeveloped, with no universally agreed definition or purpose, but a number of commonly occurring features have been identified (Levac *et al*, 2010). A scoping study is generally a precursor to further work, synthesising and analysing a wide range of material (rather than just high quality academic research) in order to provide a guide to a topic, which may take the form of a conceptual map like the model above (Davis *et al*, 2009). The guide may identify what is and is not known, and contextualise this knowledge by relating it to policy and practice, so that these can become more informed and further practically relevant research can be undertaken (Anderson *et al*, 2008). To achieve relevance, it is often desirable that the study is conducted rapidly (Arksey & O'malley, 2005), but this may be a challenge as the process is typically iterative, with the discovery of previously unappreciated aspects of a topic taking the study in new directions. Carefully designed engagement with policymakers, practitioners and researchers may help guide and speed the process, and some scoping studies also aim to develop networks across these stakeholders in order to build capacity to conduct future research.

**Figure 2:**
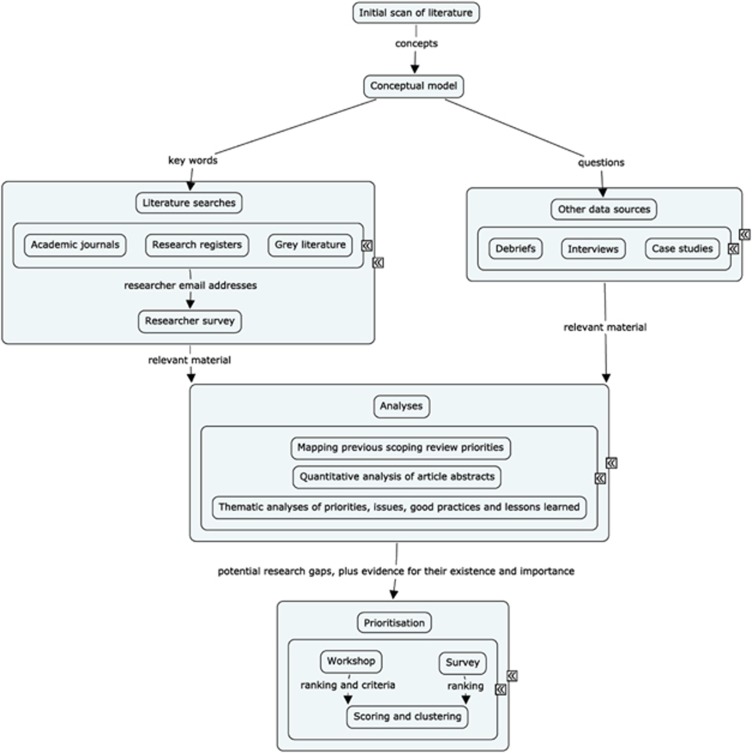
Flowchart of the research process.

Data collection took place over 12 months, starting in September 2010, and comprised various strands: a structured literature review; a survey of researchers; interviews; an exploration of debriefs of incidents and of larger case studies; and a prioritisation workshop and survey (see Figure 2). Boyd *et al* (2012) provides full methodological details. The aim was to identify not only research gaps, but also good practices and issues of concern regarding practice.

The literature review began with a keyword search of several academic databases and was followed by citation searches using Scopus and Google Scholar. Priority was given to literature reviews, and to citations focused on health or health care, on the United Kingdom and published from 2006 onwards. The recent contents pages of five key journals were also scanned, and various research registers and research funder websites were searched to locate recent and current research. Researchers identified through the literature search were asked by email to identify their most recent publications and research, and to say which topics they thought should be priorities for future research. Grey literature from the United Kingdom was found by searching the Emergency Planning College online library, NHS Evidence, the Department of Health website, the Health Protection Agency website, the U.K. Resilience and Cabinet Office website, the Centre for Public Scrutiny library of local authority scrutiny committee review reports and the Royal United Services Institute website, journal and newsbrief. All relevant citations and research project outlines were categorised using a multidimensional framework with categories for: country/area of the disaster; phase of the emergency; hazard type; research method; and various elements of preparedness. U.K.-focused research was compared with research conducted elsewhere by calculating frequencies, cross tabulations and correlations of framework categories.

Text was also extracted from documents regarding issues identified, theories developed and claims made; suggestions for improving practice and for further research; and any supporting evidence. Particular attention was paid to research priorities identified by previous scoping studies from the United States (U.S. Centers for Disease control and Prevention, 2006; Abramson *et al*, 2007; Altevogt *et al*, 2008; Kelen & Sauer, 2008; Acosta *et al*, 2009; Savoia *et al*, 2009; Yeager *et al*, 2010), as analysis suggested that their main findings would also apply to the United Kingdom. These priorities were mapped onto each other visually to produce an ordered list of nine groups of research priorities.

Semi-structured interviews were conducted with 13 people drawn from a range of U.K. stakeholder groups, including the ambulance and fire services, the Department of Health, a local council, voluntary and community organisations, and the Health Protection Agency, which provides specialist support and advice to help protect public health. Key staff from federal agencies in the United States were also interviewed. Specific issues and lessons learned were extracted from debrief reports of 20 small-scale incidents in the Greater Manchester and Lancaster areas of England. Two more detailed case studies were also examined: the 2009/2010 swine flu (H1N1) outbreak and the Cumbria floods of 2005 and 2009. Text was extracted from debrief documents and published articles, identified through a snowballing process of key informants, who were also asked to give their views on key issues, good practices and knowledge gaps.

Thematic analyses were conducted on the textual data from each of the various sources above. After integration with the nine groups identified from previous scoping reviews, 18 potential research topics and associated research questions were formulated. A workshop involving 16 people drawn from a range of stakeholder groups suggested criteria against which to assess the topics. The topics were scored against the criteria by the workshop participants, and ranked by both the participants and a further 16 people via an email survey. The stakeholder groups covered included those represented in the interviews (see above); NHS commissioning organisations and providers of acute hospital and mental health care; NHS Blood and Transplant; the Police; researchers; and the Emergency Planning College, which provides training courses. The research team developed the criteria into a more comprehensive assessment framework that considered the existence of knowledge gaps, the importance of filling those gaps and the practicality of doing so. The team then scored the research topics systematically, taking account of all of the evidence that had been uncovered by the study. The various scores and rankings were compared using average rankings, correlations between scores and average rankings, and a two-dimensional scaling analysis of the rankings.

## Findings

Four clusters of related research topics are suggested as the basis for the commissioning of future research on health-care emergency planning and management in the United Kingdom (Table 1).

### Research Cluster 1: public affected by health emergencies

Health-care emergency planning aims to protect the public's health and maintain services to treat people's illnesses. Therefore the communities to be served should be central to such planning. Yet it would appear that not enough is known about communities and how to support them, and the potential for active partnership between communities and services has not been fully realised (Ahmed *et al*, 2012).

Despite the existence of national guidance on recovery (National Recovery Working Group, 2007), which makes reference to people's health needs, recovery often seems to be the poor cousin of emergency planning and response. Yet unless recovery processes swing into action as the emergency is being dealt with, there is evidence that long-term problems can be created (Levine *et al*, 2007; Madrid *et al*, 2009). Moreover, since social, psychological and political factors can affect needs, health-care organisations should be part of an integrated recovery process that addresses the broad range of issues that concern victims, their families and the wider public. Recovery also needs to recognise that some groups are more vulnerable than others.

There are many issues relating to vulnerable populations whose health, social or economic circumstances make them more susceptible to the effects of an incident, as well as those who are made vulnerable by the incident itself. Identifying vulnerable groups can be difficult. Data relating to vulnerability is not generally held by a single agency, but needs to be garnered from a range of organisational and community sources. Information can be hard to access during an emergency, as the relevant gatekeeper both needs to be identified and persuaded to sanction release of data. Furthermore, the data quality may be uncertain, and there is an intrinsic problem that people's vulnerability varies depending on the characteristics of the incident as it proceeds.

Although there is greater recognition of the importance of communicating risk and other information to the public, and practice would appear to have improved, levels of competence across the workforce are not known. There is a lack of understanding about how communities access, generate, share, interpret and use information, particularly with the advent of new technologies and the rise of social networking tools such as Facebook and Twitter. Information generated by social networks is not quality assured and can be difficult to capture efficiently, but there are potential benefits that might be realised. These include being able to communicate with the public quickly, including monitoring public perceptions of organisational performance; capturing data to monitor the course of an incident; better targeting and analysing information through enhanced geographical awareness; and supporting communities to take action themselves.

### Research Cluster 2: inter- and intra-organisational collaboration

Constructive partnership working between agencies is important to both planning and response, and is a continued focus for policymakers and practitioners. This is particularly relevant to an NHS in the midst of a substantial reorganisation, which may dissolve existing relationships and require new relationships to be established across an increasingly diverse set of organisations, many of which are themselves new. Research is needed to better understand multi-agency working and what promotes and impedes it. For example, how does multi-agency working differ between routine operations, planned large events and major emergencies? How can the collaborative spirit engendered during incidents be built upon? It can be difficult for cross-organisational teams to have enough personal contact and familiarity to be sure that they will work effectively during an incident, despite training and exercises. In the United Kingdom, planners build relationships through planning forums, but not responders; and within organisations, there is the issue of how to develop relationships and share tacit knowledge between responders and planners.

### Cluster 3: preparing responders and their organisations

The U.K. research studies (Anathallee *et al*, 2007; Williams *et al*, 2007; Fell, 2008; Day *et al*, 2010) typically indicate shortfalls in the emergency preparedness of health-care services. Training and exercises are a major component of developing preparedness, but knowledge is lacking regarding their effectiveness. There may also be untapped potential to learn from past experiences and to make use of quality improvement methods.

Responding organisations generally debrief after any significant incident producing a report known as an After Action Report in the United States. These reports may be circulated locally, with particular recommendations and learning points being followed up, but no structured attempts have been made to collate, compare and learn from them on a larger scale. The reports represent an underused resource to improve practice, to recognise business continuity issues and also to identify emerging threats and patterns.

Training and exercises both train the participants to deal with specific features of events, and build trust and understanding. However, exercises tend to rehearse well-anticipated events that run to plan, to consider only the worst case scenario and to cover all functions and address all issues within all the allotted time. There is a danger of superficiality and of failing to consider conjunctions of events that can compromise planning assumptions.

Generally there is an issue of professional training for emergency managers and ways of sharing best practice. In the study interviews, no clear mechanisms were identified for the professional training of emergency managers other than membership of certain professional bodies, taking part in exercises and attending planning meetings. Interviewees observed that senior emergency planners may have worked their way up from being responders. This provides them with a valuable wealth of practical experience, but a paucity of relevant theory and body of evidence on which to build appropriate behavioural and management competencies.

### Cluster 4: prioritisation and decision making

The priority and the resources devoted to planning, preparedness and response are important. There is little point in knowing how to design effective training, for example, if there are insufficient funds to deliver it to staff, or if staff attendance is poor. The wider administrative and political context is influential in this regard, especially given the NHS reorganisation, associated staff ‘churn' and pressures to realise cost savings, yet little is known about exactly how wider systems impact on emergency planning and response, and about the decision-making processes of health-care leaders.

Many smaller organisations assign emergency planning to be only one part of a single manager's role. The other parts can relate to activities with continual day-to-day demands, inevitably diverting attention away from emergency planning. Similarly, although emergency planning is an NHS priority, regulatory and performance monitoring systems are not conducive to prioritising resources to deal with what may be rare events.

Organisations need senior managers who have the abilities to take effective command and control decisions during emergencies. Yet decision-making processes during crises are not fully understood (Rake, 2003; Sementelli, 2007) due to the dynamic complexity of incidents and the nature of the evolving and unknown risks that are present. This makes it difficult to provide suitable training or to incorporate assessment of the necessary abilities into recruitment processes.

## Discussion

The study's assessment of research priorities is based on three key measures that the study identified: the strength of evidence indicating the existence of a research gap; the extent to which the research would address the needs of organisations; and the extent to which the research would address the needs of communities. No individual research topic scored highly on all of these measures (Boyd *et al*, 2012), so it is prudent to set priorities at a more general level. The feasibility of research to answer specific questions should be assessed during the commissioning process.

**Figure 3:**
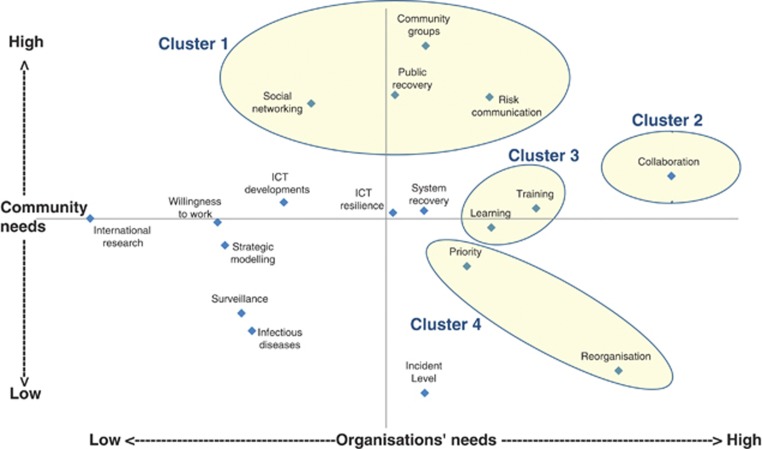
Two-dimensional scaling analysis of research topic priorities from the workshop and survey combined, showing clusters. *Note*: See Boyd *et al* (2012) for details of topics not included in the priority clusters.

Communities and organisations appear to have different views, particularly with regard to what are the most important topics needing research. Analysis of the workshop rankings (Figure 3) suggests that Cluster 1 is a high priority for communities but less so for organisations, while the remaining clusters are higher priorities for organisations than for communities, with Cluster 4 having very little resonance for communities. Some of these differences may reflect the lack of visibility to the general public of internal organisational workings, and also a lack of awareness of community concerns among emergency planners located within large organisations. This suggests the importance of trying to increase mutual understanding between organisations and communities and of planning involving a wide range of stakeholders.

There are differences between the U.K. and U.S. contexts that should be reflected in the research priorities of the two countries. Among other things, the United States has a greater incidence of extreme weather and a more complex legal situation, with both federal and state laws. Nevertheless, many of the research gaps identified by previous U.S.-focused scoping studies are also relevant to the U.K., and there would appear to be scope for collaboration between research commissioners, including the U.K. government departments and commissioners in the United States, to compare research priorities, coordinate commissioning, develop commissioning models and build capacity to conduct research, learning from the experiences of the Preparedness and Emergency Response Research Centers in the United States.

## Conclusion

Four broad research priorities have been identified for which there is good evidence of the existence of knowledge gaps that are important issues either for communities or for organisations. The findings have much in common with those of previous U.S.-focused scoping reviews, and with a recent U.K.-based review (Challen *et al*, 2012; Lee *et al*, 2012; Mackway-Jones & Carley, 2012), confirming the relative paucity of U.K.-based research. Closer cooperation between stakeholder groups within and outside the United Kingdom may be a practical way forward to developing research capacity and filling the knowledge gaps. The conceptual model presented in this paper may help by increasing understanding of emergency planning among communities, practitioners, policymakers and researchers.

## Figures and Tables

**Table 1:** Suggested future research clusters showing the level of confidence in prioritising their constituent topics and illustrative research questions^a^

*Cluster*	*Topic*	*Confidence in topic priority*^b^	*Illustrative research questions*
1. Affected public	Recovery (including long-term health impacts)	Robust	• How can social support networks be supported in the recovery phase? • What are the best interventions for addressing psychosocial health problems?
	Community involvement and vulnerable groups	Robust	• What are the relationships between community resilience and wellness following disasters? • What is the potential for active community, voluntary sector and business involvement in emergency planning and management, and how can it be developed?
	Communication and public information	Robust	• How effective are risk communication efforts during particular events? • What are the levels across the workforce of competencies in crisis risk communication?
	Social networking	Plausible	• How can social networks be monitored most effectively for intelligence on what is happening during an incident? • Can social networking be used to build trust between the authorities and the public?
2. Inter- and intra-organisational collaboration	Coordination/collaboration	Robust	• What are the factors that enable and inhibit standardisation/interoperability across organisations, including the contribution of training and exercising? • How can the collaborative spirit engendered during incidents be built upon? • How can coordination across a ‘mixed economy' of relatively autonomous health-care organisations be maintained and improved, especially during the response and recovery phases? • What is the potential for productive linking of emergency planning and management with other strategic and operational planning and management?
3. Preparing responders and their organisations	Learning and quality improvement	Robust	• What approaches are effective in facilitating learning from good practice, exercises and incidents of all sizes – locally, regionally and nationally? • What approaches (regulation or internal processes) are effective in producing continuous, sustainable quality improvement in emergency preparedness?
	Training/exercises	Robust	• What are the connections between training, competency and capability, and outcomes, for example, with regard to decision making during response? • How do we train and share best practice among emergency planners?
4. Prioritisation and decision making	Priority and resourcing	Unable to assess	• What characteristics (capabilities, capacities etc.) make for an effective emergency planner/planning function in NHS organisations? • Which factors (e.g., professional background of senior managers, political, social and administrative contexts, funding sources, targets etc.) have the greatest impact on the resources (staff, financial, equipment etc.) that organisations devote to preparedness?
	Impact of organisational change (e.g., NHS reorganisation)	Plausible	• How to maintain emergency planning and management capability and effectiveness during periods of organisational change? • How does the emergency planning system provide sufficient consistency and leadership for emergencies covering a wide geographical area?
	Social, administrative and political contexts	Plausible	• What constitutes effective and fair systems for commissioning, contracting and performance management of emergency preparedness and response (e.g., taking account of the costs and knock-on impacts of response)? • What is the impact of political imperatives on decision making with regard to emergency preparedness, response and recovery?
	Leadership and decision support systems during crises	Unable to assess	• What competencies and training are needed for NHS managers who may take on command and control roles? • How are decisions taken during emergencies, and what use is made of decision support data and of emergency plans?

a Further work would be needed to identify a specific set of priority research questions.

b Candidate topics were assessed to be ideal if they scored highly on all key measures; robust if they did not have a low score on any key measure; or plausible if they had a mixture of high and low scores. Two topics could not be assessed because they were identified after the prioritisation workshop had taken place.

## Footnotes

1 Detailed definitions of terms such as ‘recovery' that are used in emergency planning can be found in Boyd *et al* (2012).
